# The effect of concurrent use of swaddle and sucrose on the intensity of pain during venous blood sampling in neonate: a clinical trial study

**DOI:** 10.1186/s12887-022-03323-0

**Published:** 2022-05-10

**Authors:** Mahla Talebi, Seyedeh Roghayeh Jafarian Amiri, Parvin Aziznejad Roshan, Ali Zabihi, Yadollah Zahedpasha, Mohammad Chehrazi

**Affiliations:** 1 Student Research Committee, Babol University of Medical Sciences, Babol, Iran; 2 Department of Medical & Surgical Nursing, School of Nursing & Midwifery, Babol University of Medical Sciences, Babol, I.R Iran; 3 Amirkola Children’s Non-Communicable Disease Research Center, Health Research Center, Babol University of Medical Sciences, Babol, I.R Iran; 4 Social Determinants of Health Research Center, Health Research Institute, Babol University of Medical Sciences, Babol, I.R Iran; 5 Department of Pediatrics, School of Medicine, Non-Communicable Pediatric Disease Research Center Health Research Institute Amirkola Hospital Babol University of Medical Sciences, Babol, Iran; 6 Department of Biostatistics and Epidemiology, School of Health, Babol University of Medical Sciences, Babol, I.R Iran

**Keywords:** Sucrose, Swaddle, Blood sampling, Pain management, Infant, Clinical trial

## Abstract

**Background & objective:**

Infants undergo painful procedures while receiving care and treatment. Blood sampling is the most common painful procedure for infants. Pain control plays a significant role in preventing unwanted physical and psychological effects. Therefore, this study aimed to investigate the effect of concurrent use of swaddle and sucrose taste on the pain intensity during venous blood sampling in neonates.

**Methods:**

In this clinical trial study, 60 infants admitted to the neonatal ward of Amirkola Hospital were randomly divided into four groups of 15 patients. In the first group, the infants were swaddled before blood sampling. In the second group, sucrose was administered to infants. In the third group, the neonates were swaddled and given sucrose simultaneously, and in the fourth group (control), blood sampling was performed routinely. PIPP pain scale and demographic questionnaire were used to collect the data. Data analysis was performed using SPSS23.

**Results:**

The results showed a significant difference between the mean pain intensity in neonates in the sucrose-swaddle group (4.53 ± 1.30) and the sucrose (7.73 ± 2.73), swaddle (9.86 ± 33.33), and control (12.13 ± 2.06) groups during blood sampling (*P* < 0.001). Besides, after blood sampling, there was a significant difference between the mean pain intensity in neonates in the sucrose-swaddle group (4.33 ± 1.23) and the sucrose (8.13 ± 2.66), swaddle (7.73 ± 2.78), and control (10.00. ± 1.96) groups (*P* < 0.001).

**Conclusion:**

The present study showed that pain severity during and after venous blood sampling was lower in the swaddle-sucrose group than in other groups. Therefore, it is recommended that the combined method of swaddle-sucrose be used in infants as a better pain reliever when intravenous blood sampling is performed.

## Introduction

The neonatal period includes 28 days after birth [1]. According to the World Health Organization (WHO), 130 million neonates are born each year [2]. Besides, according to the statistics of the Ministry of Health of Iran, about 1,200,000 neonates are born annually [3]. The positive and negative environmental effects profoundly influence brain development during the neonatal period and create future health outcomes [4].

Pain is an unpleasant feeling experienced throughout a person’s life and can be exacerbated by an illness or specific procedures in the hospital. The American Pain Association (1995) has labeled pain as the fifth vital sign and emphasizes the importance of repeated pain assessment [5].

Pain in neonates is crucial since the sensory area is the most active area of ​​their brain, and the pain transmission pathway is fully developed; however, its inhibitory systems are not well developed [6]. Newborns in the neonatal ward are regularly exposed to painful experiences and have the right to receive safe, efficient, and effective pain relief [7]. According to the evidence, painful stimuli affect early life experiences as well as short- and long-term life outcomes, including sleep disorders, heart rate, blood pressure, oxygenation, gastrointestinal motility, infant learning ability, hearing, nutrition, and growth [8].

All neonates undergo at least one painful procedure during the first few days of their life, such as screening the neonate with heel blood for bilirubin [9]. Most procedures performed by physicians and nurses in the neonatal intensive care unit are painful and unbearable for the neonate [10]. Research has shown that neonates in the intensive care unit experience 7.5 to 17.3 painful procedures [11].

Pain can be relieved through pharmacological and non-pharmacological methods [12]. In order to reduce the use of multiple medications to relieve pain and minimize adverse effects on immature neonatal systems [13], the nurse may use non-pharmacological methods such as sensory stimulation (swaddling, shaking, aromatherapy, non-nutritious sucking, and music), nutritious (sweet oral solutions) and maternal interventions (mother’s scent and voice, breastfeeding, skin-to-skin contact, kangaroo care) as effective strategies to reduce pain in neonates [14].

It is preferable to use pharmaceutical methods combined with non-pharmacological methods. Such methods are valuable alternatives for controlling pain when performing painful, invasive procedures in newborns [15].

Oral sucrose is the most well-known non-pharmacological interventions, widely used in clinics to relieve pain in newborns. A systematic review (2016) showed the positive effects of sucrose in the short term to reduce the acute pain response caused by painful procedures in neonates [16].

Swaddling is one of the non-pharmacological interventions that has been overlooked for years and is being revived [17]. It is a method that reduces pain in premature and term infants, promotes the development of the central body system [18], positively affects infants, reduces physiological behaviors and infant crying, and improves infant sleep quality [19].

Some evidence has shown that sucrose, in combination with other non-pharmacological interventions, is more effective in relieving pain than sucrose alone [16]. A combination of two or more non-pharmacological interventions may be more efficient than using one method of pain relief [20].

Some moderate-quality evidence shows that sucrose in combination with other non-pharmacological interventions such as non-nutritive sucking is more effective than sucrose alone; however, further research is needed [16]. Due to their unique position in neonatal care, nurses need to be aware of research on non-pharmacological methods of pain control and routinely begin pain relief with non-pharmacological methods [21]. In addition, swaddle-sucrose administration is a low-cost method with high applicability; therefore, this study was conducted to investigate the effect of the combined use of swaddling and tasting sucrose during venous blood sampling on neonatal pain intensity.

## Materials and methods

This clinical trial was conducted in 2021 on 60 term neonates admitted to the neonatal ward of Amirkola Children’s Hospital in Babol who met the inclusion criteria after obtaining permission from the ethics committee of Babol University of Medical Sciences with the code IR.MUBABOL.REC.1399.256 and informed consent. Primary sampling was performed through the convenient method. Afterward, the neonates were assigned to four groups of control, sucrose, swaddle, and swaddle combined with sucrose, based on the random allocation method. After applying the inclusion and exclusion criteria, the study subjects were assigned into groups using the permuted block randomization method. The randomization unit included individuals, and the block size was 4. The intervention group was repeated once in each block. To conceal random allocation, a list generated by a statistician was used. The assigned group of each subject had a specific code that was a combination of numbers and letters with no specific order. This list was not provided to the principal of the study (researcher) and the person responsible for the allocation. After sampling, the individuals were assigned to the groups based on the allocation list provided by the statistician.

Inclusion criteria included: Term infants with a gestational age of 42–37 weeks, with Apgar scores above 7 in 5 minutes, stability of vital signs before sampling, and no pain experienced due to previous blood sampling (painful procedure) for at least the last 6 hours. Exclusion criteria included term infants with congenital or genetic abnormalities, infants exposed to antidepressants and anticonvulsants by the mother during pregnancy, term infants exposed to surgery who received anesthesia, and infants who received sedatives for 12 hours before sampling.

The present study was registered with the IRCT20200913048704N2 number on the Iranian clinical trial site on 13.07.2021.

Utilizing the formula of comparing two independent groups means (with the equal assignment to each group) and considering 95% confidence level, 90% study power, type 1 error of 5%, and mean and standard deviation of 13.3 ± 1.6 for the control group and 10.1 ± 2.0 for the sucrose group, the sample size was calculated as 12 individuals for each group based on Gao’s study [22]. It should be noted that type I error was adjusted by the Bonferroni method due to multiple comparisons. After calculating 20% ​​sample attrition during the study, 60 eligible infants were equally assigned to 4 groups (15 in each group). Sampling lasted for four months.

This study setting was the neonatal ward of Amirkala Hospital in Babol. In this study, the primary outcome was “pain intensity during and after venous blood sampling in infants,” and the secondary outcome was “physiological changes in infants during and after venous blood sampling.”

Sucrose was administered as follows: 0.2 ml/kg of standard 24% sucrose was dumped into the infant’s mouth with a 1 ml needleless syringe 2 minutes before venous blood sampling. It should be noted that before venous blood sampling, no pacifier or other intervention was used to calm the neonate.

Swaddling the neonate was performed as follows: the neonate was laid on a triangular cloth on a flat surface with no clothes and only a diaper. At first, one side of the sheet, then the bottom, and finally, the other side was folded up over the neonate. In this method, called Frog Flexible, the neonate is able to move the pelvic joints effortlessly, and the arms are bent and placed along the line under the chin, similar to the neonate’s position in the mother’s uterine. The infant’s hand was removed from the swaddle after 10 minutes of swaddling (checked on the stopwatch), and venous sampling was performed by a nurse with more than 18 years of experience in the neonatal ward for all infants using needle NO. 24–26. The blood was collected dropwise without using a syringe. After placing a small dressing on the blood collection site, the neonate’s hand was placed in the swaddle again, and the swaddle was maintained for up to 2 minutes after collecting blood.

In the third group, the infants received sucrose and swaddle simultaneously. In the fourth or control group, the infants received no (non) pharmacological interventions during the painful intervention. Before taking blood samples from each neonate, the researcher completed the first section of the questionnaire. In all four groups, physiological parameters (heart rate, arterial oxygen saturation) were recorded by the cardiorespiratory monitor model Saadat/Alborz-B9 in three stages: before, during, and after blood sampling (Intended for 30 seconds) and calculated as mean and standard deviation. In order to record the behavioral response, video recording was performed by the assistant researcher from two minutes before sampling to two minutes after it, using the mobile phone camera. The videos were then coded and interpreted in three stages (two minutes before, during, and two minutes after blood sampling) by a trained research assistant who was unaware of groupings and statistical analysis.

Data collection tools included a demographic questionnaire and neonatal pain measurement scale (PIPP).^1^ Maternal and neonatal demographic information was obtained from the maternal and neonatal hospital records.

The Neonatal Pain Measurement Scale (PIPP) has the desired validity and reliability of 0.93–0.93 [23, 24]. This tool contains seven useful items for both groups of term and premature neonates [23], each with four options. Three indicators of this instrument include neonate behavioral tests (raising eyebrows, squeezing the eyes, and grooving along the laugh line), two indicators include physiological tests (changes in heart rate and oxygen saturation), and two other indicators include background factors (gestational age and behavioral status). Each clause contains scores from zero to 3; consequently, the overall score of the instrument is 0–21 (minimum score = 0, maximum score = 21). A score of 0–6 indicates minimal pain or analgesia. A score of 7–12 indicates mild to moderate pain that requires non-pharmacological interventions, and scores above 12 indicate moderate to severe pain that requires medication and comfort. In this study, the PIPP score was recorded 2 minutes before, during, and 2 minutes after blood sampling (the observation time for recording the PIPP score was 30 seconds).

The collected data were entered in SPSS software version 23. For continuous variables, data are presented as mean ± SD. Split-plot repeated measure ANOVA was used to compare mean continuous variables across the four intervention groups and to assess time trend responses while controlling for baseline measurement. Afterward, either Bonferroni (in case of homogeneity of variances) or Dunnett T3 (in case of heterogeneity of variances) post hoc test was performed. We used Mauchly’s test of sphericity as a fundamental assumption for repeated measures ANOVA. If the assumption was violated, a Greenhouse-Geisser correction was applied. The significance level was considered less than 0.05.

## Results

In this study, 60 term neonates were studied, of whom 32 (53.3%) were girls, and 28 (46.7%) were boys. Mean gestational age and mean weight was 38.65 ± 0.7 weeks and 3405.67 ± 410.89 g, respectively. Forty-one deliveries (68.3%) were cesarean section, and 19(31.7%) were vaginal. Neonates did not significantly differ in terms of sex, weight, height, head circumference, type of delivery, and gestational age (*p* > 0.0 5)

The results showed that changes in heart rate were significantly different only during blood sampling (*P* = 0.009). Pairwise comparisons of the groups showed that neonates’ heart rate in the sucrose swaddle and control groups was significantly different during blood sampling (*P* = 0.006) (Table 1).

**Table 1:** Comparison of neonatal heart rate between and within study groups in baseline, during, and after blood sampling

Stages	Baseline	During blood sampling	After blood sampling
Groups	Mean ± SD	Mean ± SD	Mean ± SD
Sucrose (*n* = 15)	134.00 ± 8.58	149.06 ± 10.03	141.86 ± 14.47
Swaddle (*n* = 15)	133.86 ± 11.52	147.33 ± 17.43	138.26 ± 17.33
sucrose swaddle (*n* = 15)	131.86 ± 11.02	135.80 ± 14.24^a^	133.80 ± 11.43
Control (*n* = 15)	128.26 ± 4.83	146.06 ± 19.88	133.40 ± 9.12
*P* value	0.09	0.009	0.48

^a^ significantly different compared to the control group

Table 2 shows that during and after blood sampling, changes in the percentage of neonates’ arterial oxygen saturation among the study groups were significantly different. Pairwise comparisons showed that the percentage of neonates’ arterial oxygen saturation in the sucrose-swaddle group, control group (*P* = 0.01), and sucrose and control group was significantly different (*P* = 0.04) during blood sampling. In addition, after blood sampling, the percentage of arterial oxygen saturation of neonates in the sucrose-swaddle and control groups (*P* = 0.008) was significantly different.

**Table 2:** Comparison of neonatal arterial oxygen saturation percentage between and within study groups in baseline, during, and after blood sampling

Stages	Baseline	During blood sampling	After blood sampling
Groups	Mean ± SD	Mean ± SD	Mean ± SD
Sucrose (*n* = 15)	97.13 ± 2.32	95.46 ± 2.8 ^a^	95.80 ± 2.93
Swaddle (*n* = 15)	97.80 ± 1.81	94.53 ± 2.68	95.80 ± 2.46
sucrose swaddle(*n* = 15)	97.93 ± 1.79	96.80 ± 1.97^a^	97.46 ± 1.95 ^a^
Control (*n* = 15)	95.73 ± 2.15	91.33 ± 4.90	92.86 ± 3.13
*P* value	0.055	0.007	0.009

^a^significantly different compared to the control group

The results showed that during and after blood sampling, changes in neonatal pain in the study groups were significantly different (*P* = 0.001). Pairwise comparisons showed that during blood sampling, the pain intensity of neonates in the sucrose swaddle and control groups (*P* = 0.001), sucrose and control groups (*P* = 0.001), and swaddle and control groups (*P* = 0.001) was significantly different. The results also showed that neonates’ pain in the control and other groups was significantly different after blood sampling (*P* = 0.001) (Table 3).

**Table 3:** Comparison of neonatal pain between and within study groups in baseline, during, and after blood sampling

Stages	Baseline	During blood sampling	After blood sampling
Groups	Mean ± SD	Mean ± SD	Mean ± SD
Sucrose (*n* = 15)	4.26 ± 1.71	7.73 ± 2.73 ^a^	8.13 ± 2.66 ^a^
Swaddle (*n* = 15)	4.53 ± 2.35	9.86 ± 3.33 ^a^	7.73 ± 2.78 ^a^
sucrose swaddle (*n* = 15)	3.46 ± 1.24	4.53 ± 1.30^a, b, c^	4.33 ± 1.23 ^a, b, c^
Control (*n* = 15)	2.80 ± 1.08	12.13 ± 2.06	10.00 ± 1.96
*P* value	0.059	0.001	0.001

^a^ significantly different compared to the control group, ^b^ significantly different compared to the sucrose group, ^c^ significantly different compared to the swaddle group

**Fig. 1:**
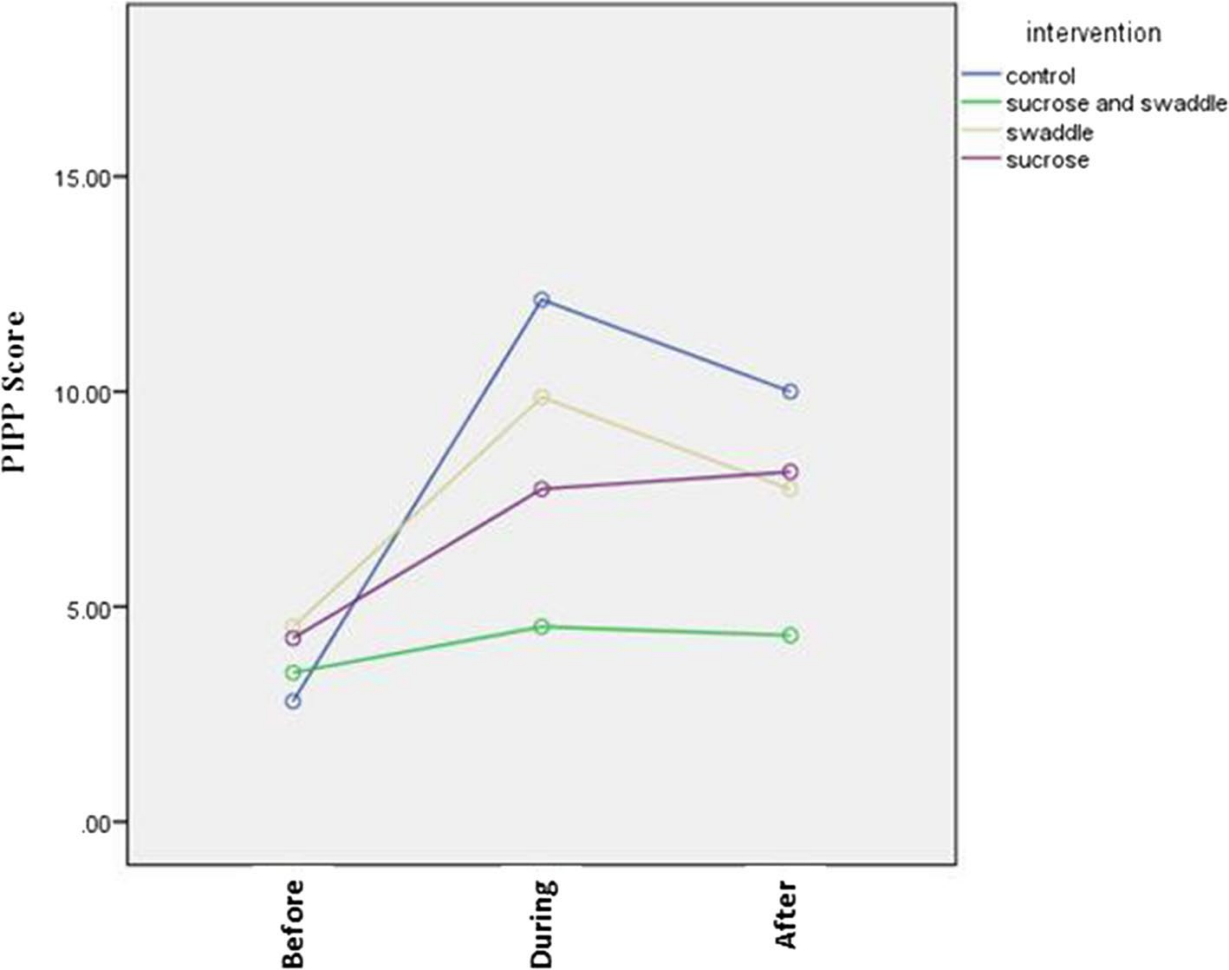
Comparison of neonatal pain changes before, during, and after blood sampling in study groups

According to Table 3 and Fig. 1, the trend of pain changes in the sucrose swaddle group was less than in other groups.

## Discussion

This study showed that the changes in neonatal pain during and after blood sampling were significantly different between the studied groups. In other words, neonatal pain intensity was significantly different during blood sampling in the sucrose-swaddle and control groups, sucrose and control groups, and swaddle and control groups. Moreover, after blood sampling, neonates’ pain intensity was significantly different in the control group and each of the other groups. According to the data from this study, the trend of pain changes in the sucrose swaddle group was less than in other groups. In other words, the combined use of sucrose and swaddle was more effective in reducing the pain caused by blood sampling than sucrose or swaddle interventions alone. In the study by Leng et al. (2016), newborns who received three non-pharmacological interventions of sucrose, swaddle, and non-nutritive sucking simultaneously during heel stick procedures experienced the least pain compared to the other groups. Moreover, newborns who received sucrose and swaddle interventions or sucrose and non-nutritive sucking simultaneously showed less pain than infants who used only sucrose [25].

In a study by McNair et al. (2019), using combined non-pharmacological interventions such as sucrose, swaddle, non-nutritive sucking, breastfeeding, and the like during painful procedures in infants is recommended to reduce pain [26]. In general, sucrose with a concentration of 24 to 25% has an analgesic and sedative effect on the neonate, and its mechanism of action is thought to involve activating the endogenous opioid system through taste, which acts as an opioid analgesic [27]. Furthermore, since the swaddle has multidimensional effects and improves the baby’s sleep by stimulating the sense of touch, warming, and distraction, it may optimally control the pain [28]. Therefore, the combined use of these interventions during blood sampling can intensify the analgesic and sedative effects.

The study by Viana et al. (2020) showed that newborns who received music and swaddle interventions simultaneously experienced less pain and lower heart rate changes during venipuncture at all stages of the intervention [29]. Another study by Yousef et al. (2019) found that although oral sucrose or pacifier alone reduced the mean pain score in preterm neonates during painful procedures, combined use of a pacifier and oral sucrose showed improvement in reducing pain than using each of them alone [30]. Furthermore, in the study by Gao et al. in 2018, it was shown that the concurrent use of non-pharmacological interventions (non-nutritive sucking and sucrose) in pain relief during painful procedures was more effective than using each of those interventions alone [22]. In general, according to the findings of the present and other studies, it can be concluded that by using a combined non-pharmacological intervention of swaddling and sucrose administration, pain in neonates can be reduced before blood sampling. The results of this study can be interpreted as follows: the sedative effects of a combination of swaddling and sucrose interventions are greater than each when used alone. Given that swaddling is convenient and inexpensive care, to make infants more comfortable while performing painful procedures, the simultaneous use of these two interventions is recommended.

The present study results showed that changes in heart rate were significantly different only during blood sampling between the studied groups, and the heart rate of neonates in the sucrose-swaddle group and the control group was significantly different. In other words, 2 minutes after blood sampling, the changes in the neonates’ heart rate decreased and gradually returned to the state before blood sampling. This decrease in heart rate was similar in all four groups for 2 minutes after blood sampling. It may be because the changes in heart rate caused by painful procedures are short-term. In other words, the pain peaks in the very first moments, and the pain perception decreases 2 minutes after the injection. Therefore, pain prevention in painful procedures such as injections can play an important role in reducing infants’ heart rate. Similar to our study, in other studies, non-pharmacological interventions when performing painful procedures led to a reduction in neonatal heart rate and pain perception in the intervention groups compared to the control group [25, 31, 32].

According to the statistical data of this study, the neonates of the sucrose-swaddle group had the highest percentage of arterial blood oxygen saturation during and after blood sampling, and the changes were less than the neonates of other groups. A study by Zeraati et al. (2014) showed that non-pharmacological interventions cause a smaller reduction in oxygen saturation during eye examination [33]. Moreover, in the study by Gao et al. (2018), combined non-pharmacological interventions (sucrose and non-nutrient sucking) before blood collection from the heel led to a decrease in heart rate and increased oxygen saturation [22], which is consistent with the present study. According to another study, non-pharmacological interventions, in addition to reducing pain, can reduce its physiological changes [34]. However, there is not always a reduction in physiological changes after non-pharmacological interventions [25].

One of the strengths of the present study is the randomized clinical trial design with a control group and the approach of applying combined non-pharmacological pain management in neonates, which has rarely been done. One of the limitations of this study was the device and equipment alarms, such as the ventilator, respirators, monitors, crowding of the ward staff and students, and the ward noise in the morning shift, which may affect the infant’s physiological parameters. In order to better understand the effect of combined non-pharmacological pain management, more research with larger sample sizes is needed.

## Conclusion

The present study provides clinical evidence that the effectiveness of combined non-pharmacological pain management in controlling pain is greater than using interventions alone when performing more painful procedures. Swaddle-sucrose non-pharmacological pain management is convenient, effective, practical, and low-cost. Therefore, in order to better manage and relieve pain when venous blood sampling is performed in the neonatal ward, it is recommended that the combined sucrose-swaddle method be used instead of routine methods such as using sucrose alone or without non-pharmacological intervention.

## Data Availability

The datasets generated and/or analyzed during the current study are not publicly available due [individual privacy could be compromised] but are available from the corresponding author on reasonable request.

## Abbreviations

PIPP: Premature Infant Pain Profile
SD: Standard deviation

## Footnotes

1 premature infant pain profile
